# Comparison and fusion prediction model for lung adenocarcinoma with micropapillary and solid pattern using clinicoradiographic, radiomics and deep learning features

**DOI:** 10.1038/s41598-023-36409-5

**Published:** 2023-06-08

**Authors:** Fen Wang, Cheng-Long Wang, Yin-Qiao Yi, Teng Zhang, Yan Zhong, Jia-Jia Zhu, Hai Li, Guang Yang, Tong-Fu Yu, Hai Xu, Mei Yuan

**Affiliations:** 1 Department of Medical Imaging Center, The Affiliated Huaian NO.1 People’s Hospital of Nanjing Medical University, No. 1 West Huanghe Road, Huaian, 223300 China; 2 Department of Radiology, The First Affiliated Hospital of Nanjing Medical University, 300 GuangZhou Road, Nanjing, 210029 China; 3 Shanghai Key Laboratory of Magnetic Resonance, East China Normal University, Shanghai, 200062 China; 4 Department of Radiology, Jiangsu Province Official Hospital, Nanjing, 210024 China; 5 Department of Pathology, The First Affiliated Hospital of Nanjing Medical University, Nanjing, 210029 China; 6 Department of Radiology, the First Affiliated Hospital of Nanjing Medical University, Jiangsu Province, 300, Guangzhou Road, Nanjing, 210029 China

**Keywords:** Cancer, Medical research

## Abstract

To investigate whether the combination scheme of deep learning score (DL-score) and radiomics can improve preoperative diagnosis in the presence of micropapillary/solid (MPP/SOL) patterns in lung adenocarcinoma (ADC). A retrospective cohort of 514 confirmed pathologically lung ADC in 512 patients after surgery was enrolled. The clinicoradiographic model (model 1) and radiomics model (model 2) were developed with logistic regression. The deep learning model (model 3) was constructed based on the deep learning score (DL-score). The combine model (model 4) was based on DL-score and R-score and clinicoradiographic variables. The performance of these models was evaluated with area under the receiver operating characteristic curve (AUC) and compared using DeLong's test internally and externally. The prediction nomogram was plotted, and clinical utility depicted with decision curve. The performance of model 1, model 2, model 3 and model 4 was supported by AUCs of 0.848, 0.896, 0.906, 0.921 in the Internal validation set, that of 0.700, 0.801, 0.730, 0.827 in external validation set, respectively. These models existed statistical significance in internal validation (model 4 vs model 3, P = 0.016; model 4 vs model 1, P = 0.009, respectively) and external validation (model 4 vs model 2, P = 0.036; model 4 vs model 3, P = 0.047; model 4 vs model 1, P = 0.016, respectively). The decision curve analysis (DCA) demonstrated that model 4 predicting the lung ADC with MPP/SOL structure would be more beneficial than the model 1and model 3 but comparable with the model 2. The combined model can improve preoperative diagnosis in the presence of MPP/SOL pattern in lung ADC in clinical practice.

## Introduction

Lung cancer is the leading cause of cancer mortality worldwide, and adenocarcinoma (ADC) accounts for almost half of all lung cancers^1^. Surgical resection, such as curative-intent surgery, has been shown to be an efficient therapeutic option for early-stage lung ADC. However, tumors with micropapillary/solid (MPP/SOL) patterns, even with a small amount, have been observed to have an increased risk of postoperative recurrence or metastasis^2–5^. Therefore, preoperative diagnosis of lung ADC with MPP/SOL pattern is critical for developing a suitable therapeutic scheme.

A variety of invasive and non-invasive techniques have been used for the preoperative assessment of lung ADC with MPP/SOL patterns. A novel invasive method^6^ to support the preoperative scheme has been used in clinical practice to rapidly diagnose lung ADC with MPP/SOL pattern. Preoperative histologic examination using CT-guided percutaneous biopsy cannot accurately represent the entire heterogeneous tumor^7^. Numerous studies have recently demonstrated that radiomics techniques are noninvasive approaches for predicting lung cancer based on MPP/SOL patterns via high-dimensional quantitative feature extraction from CT imaging modality^8–13^. Wang et al.^10^ proposed a method combining radiomics and deep learning (RDL) to discriminate between micropapillary and solid patterns in lung ADC expressed as ground-glass opacification. The merged radiomics and deep learning (RDL) method outperformed the radiomics method or deep learning alone, with an accuracy of 0.913 in the derivation dataset and 0.966 in the independent validation dataset. Chen et al.^11^ found that combing quantitative image analysis with near-pure radiomics values, the presence of micropapillary and solid components could be predicted with 90.00 ± 0.00% sensitivity and 77.12 ± 2.67% specificity for the derivation cohort, and with 100% and 95.35% sensitivity and specificity, respectively, for the external validation cohort. He et al.^12^ developed four radiomics-based models to predict the presence of micropapillary or solid pattern in 461 lung ADC, achieving comparable prediction performance in terms of area under the curve (AUC) in Internal validation vs external validation using a generalized linear model (0.74 vs.0.70); Naïve Bayes, (0.75 vs.0.72); SVM (Support vector machine) (0.73 vs.0.73) and random forest (0.72 vs.0.69), respectively. Park et al.^13^ developed a radiomics approach for differentiating the predominant subtype-based prognostic groups of lung adenocarcinoma (group 0: lepidic; group 1: acinar/papillary; group 2: solid/micropapillary) using CT radiomics features, achieving AUCs of 0.892 and 0.895 on the development and validation sets, respectively. Gao et al.^14^ proposed a semi-supervised learning framework that applies semi-supervised learning method to detect micropapillary adenocarcinoma, the semi-supervised learning method achieves a precision of 0.775 and recall of 0.896, which is better than supervised learning (a precision of 0.762 and recall of 0.884). Chen et al.^15^ investigated a new model of incorporating solid attenuation component masks with deep learning in the prediction of lung ADC with MPP/SOL patterns(the components of MPP/SOL > 1%) to optimize surgical strategy preoperatively with AUCs of 0.91 for the cross-validation, and 0.93 for external validation, significant better than another 3 independent model. Each of the preceding studies attempted to classify lung ADC with micropapillary and solid histological patterns using unique dataset and special non-invasive technology merely with the radiomics approaches or combined technology of radiomics and deep learning or clinic approaches to validate lung ADC with MPP/SOL patterns.

Xia and Hirsch et al.^16,17^ suggested that additional research be conducted on combining additional data, such as clinical data, to improve the performance of the merged scheme in predicting the invasiveness risk of Stage I Lung adenocarcinoma. Based on their analysis, we hypothesized a new scheme of combining computed-derived radiomics features and deep learning with clinicoradiographic variables for preoperative diagnosis of lung ADC with micropapillary and solid patterns.

## Materials and methods

### Patients

This respective multicohort study was approved by the Institutional Review Board of The First Affiliated Hospital of Nanjing Medical University (Permit Number: 2021-SRFA-238) and the Institutional Review Board of the Affiliated Huaian NO.1 People’s Hospital of Nanjing Medical University (Permit Number: 2022-0451-01), respectively. And the requirement of written informed consent was waived because that all data sources applied (demography, laboratory records, and chest CT) were previously available and analyzed anonymously and de-identified using study identifier before reading by the radiologists, model development, internally and externally validation. The need to obtain informed consent from all participants was waived by the Institutional Review Board of The First Affiliated Hospital of Nanjing Medical University and the Institutional Review Board of The Affiliated Huaian NO.1 People’s Hospital of Nanjing Medical University. All methods were performed in accordance with the relevant guidelines and regulations.

The study reviewed 2567 cases undergoing CT scans for preoperative assessment from April 2016 to October 2019. All cases of lung ADC were histologically proven and recorded in our hospital database. The inclusion criteria were as follows: (a) no prior history of other cancer; (b) no prior history of radiation therapy or chemotherapy before chest surgery; (c) pathologically confirmed to be ADC and without any variant subtypes (colloid, mucinous, fatal adenocarcinoma, etc.); (d) CT images with thin section (1.5/1.0 mm) quality were adequate for analysis; (e) clinical and imaging data for this study were obtained from the medical records database; (f) the patients with tumor was no more than stage III A. Patients were excluded for one of the following reasons: (a) no preoperative CT scan at our institution (n = 154); (b) distal metastasis (n = 4); (c) history of radiation and chemotherapy before scanning (n = 19); (d) unsatisfactory imaging quality due to respiratory artifact during examination (n = 60); (e) insufficient laboratory examination data (n = 296), (f) minimally invasive adenocarcinoma (n = 648).

**Figure 1:**
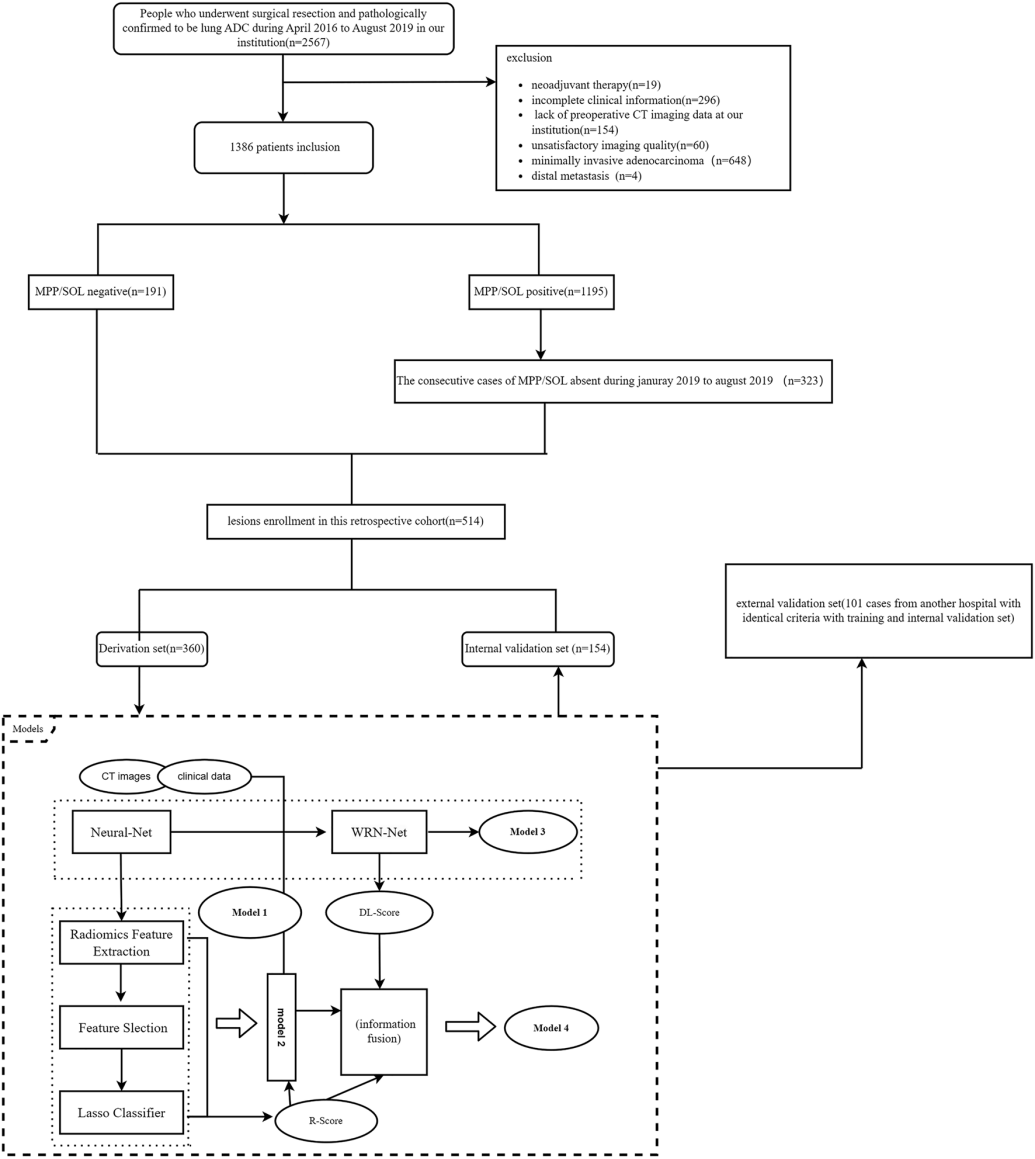
Workflow of our study show the pathway of patient inclusion and exclusion and flowchart of proposed models.

Finally, of 514 pulmonary lesions were recorded in 512 patients (males: females, 228:284; mean age ± standard deviation SD, 59.3 ± 10.1 years; range 26–82 years) in our institution. Of these, two lesions were detected in 2 and one in 510 patients. The derivation cohort had 360 cases, including 134 MPP/SOL positive and 226 MPP/SOL negative. Additionally, 154 cases assigned as the independent internal validation cohort were consisted of 57 MPP/SOL positive and 97 MPP/SOL negative. We attempted to search data for external validation on the public dataset collected in TCIA (https://www.cancerimagingarchive.net/), but mainly because of the lack of concrete pathological results. Therefore, we found another 101 cases (male: female, 48:53; mean age, 60.7 ± 9.4 years; range 31–75 years) from another hospital as the external validation cohort. Workflow of our study is shown in Fig. 1.

### Diagnostic criteria

The pathologist (author #7, with 6 years of experience in pathological diagnosis) evaluated all specimens according to the current 2015 World Health Organization (WHO) classification of lung tumors as heralded by IASLC (International Association for the Study of Lung Cancer)/ATS (American Thoracic Society)/ERS (European Respiratory Society). The semi-quantitatively assessment of five histologic patterns of lung ADC is based on the percentage of each tumor slides′ components in 5% increments. According to the description of the histological growth pattern and histologic classification^4,18^, the enrolled cases are assigned as follows: MPP/SOL Lung ADC with lepidic pattern, acinar pattern, or papillary pattern is included in the absence group(MPP/SOL−, n = 323). MPP/SOL lung ADC with micropapillary pattern or/and solid pattern is included in the positive group (MPP/SOL+, n = 191). Lesions were classified randomly as derivation set with MPP/SOL positive (n = 134) and without MPP/SOL (n = 226). The total of 154 lesions (MPP/SOL positive/negative:57/97) from our hospital was assigned to the internal validation set. Another 101 cases (MPP/SOL positive/negative:41/60) from another hospital was identified as the external validation set.

### Imaging protocols

Imaging acquisition was performed at our institution using an unenhanced CT scanner with 64-slice detector (SIEMENS SOMATOM Force; SIEMENS Definition AS+; GE MEDICAL SYSTEMS Revolution) and 128-slice detector (Philips iCT 256). All CT scans were performed in the supine position from the thoracic inlet to the margin of the kidney. All image protocol shared the following parameters: slice thickness,1.5 mm; tube voltage, 100–120 kVp; tube current, 80–300 mAs; matrix size, 256 × 256; and field of view, 252 × 252 mm, voxel size, 1 × 1 × 1 mm. A high-resolution algorithm was used to continuously reconstruct all images with a thin-section of 1.5/1.0 mm intervals. Another institution enrolled cased were scanned by SIEMENS SOMATOM Force 64-row CT machine with following scanner parameters: Tube voltage 100–120 kV, tube current 60–100 mA. Matrix, 256 × 256; and field of view, 252 × 252 mm. All images had lung windows (width, 1500 HU, level, 600 HU) and mediastinal windows (width, 350 HU, level, 50 HU).

### Establishing the clinicoradiographic model

The CT images were evaluated independently by two radiologists (author #1 and author #4, 3 and 8 years of experience in thoracic imaging diagnosis, respectively). High-resolution CT (HRCT) images (qualitative and quantitative) using to evaluate morphological features. The morphological features such as maximum tumor diameter consolidation/tumor diameter (C/T) ratio calculated by twice decoding, density (pure ground glass, mixed ground glass or solid), margin (lobulation and spiculation: absent or present), lung-tumor interface(clear or blur); internal manifestation (cavity, calcification, and honeycomb sign: absent or present), pleural tag and indentation (absent or present)^19^, change in the vessels (absent or present), relation to bronchus (absent or present). The term "change in the vessel" was defined as the formation of a collection of blood vessels adjacent to the tumor, and multi-planar reconstruction (MPR) was used to determine whether the change was present. According to Qiang et al.^20^, the tumor-bronchus relationship assessment criteria have been modified to include visual evaluation of a positive manifestation of tumor-bronchus as bronchus extension into the lesion with a tapered lumen and interruption, or bronchus abrupt obstruction on the margin of the lesion. The clinic data involves gender, age, smoking history, family history and serum biomarkers including carcinoembryonic antigen (CEA) value, neuro-specific enolase (NSE) value, cytokeratin fragment 21-1(CYFRA21-1) value, which are recorded and categorized according to the level of 4.70 ng/ml, 16.30 ng/ml and 3.30 ng/ml, respectively. Kappa values and intra-class correlation coefficients (ICCs) were calculated to assess the consistency of the two authors′ radiology evaluations.

Univariate analyses were used to determine the differences between MPP/SOL negative and positive groups on the derivation set. Logistic regression analysis was adopted to build clinicoradiographic model.

### Radiomics model built

The radiomics model was built including four steps, that is, volumes of interest (VOI) segmentation, radiomics features extraction, feature selection and radiomics signature establishment and assessment. The VOIs encompass the entire tumor information. Semi-automatic contouring was performed in thin-section (1.0/1.5 mm) CT images with in-house software (MULTILABEL; ECNU, Shanghai, China). The semiautomatic identification of the VOI of the lesions relies on radiologists to locate the lesion, and then were implemented with a cooperation of semi-automatic segmentation thresholding algorithm and a manual adjustment approach of delineation on every section of the CT scans, containing two major steps as reported in forepassed literatures^15^. The initial segmentation is followed by the step of removal of surrounding vessel, bronchus, and calcification. The radiologists with 3 years of thoracic diagnosis experience (authors #1) and another one with 8 years of experience (author#4) blinded to experimental design reviewed the image and annotated VOIs avoiding necrosis, calcification, vascular structure, etc^8^. Once the true tumor boundary cannot be deduced precisely from the image, another radiologist with 10 years of experience in chest CT interpretation reviewed and confirmed the delineation of the lesion. The disagreements would be resolved by the observer′s investigation and comprehensive assessment. To ensure the stability of the radiomics feature extraction, the VOI of each lesion was drawn twice by each of two independent radiologists. The radiologists (author#1) annotated the VOIs of 60 cases randomly selected from the study after 3 months. The intra- and inter-class correlation coefficients were calculated after the segmentation. VOI segmentation information was converted to the NII format, followed by the features extraction with the aid of FeAture Explorer Pro (FAE Pro, V0. 3.7, (https://github.com/salan668/FAE.git) on Python (3.7.6)^21^. The process of image clip is making that pixel values less than 5% and more than 95% are controlled at 5% and 95% respectively to remove the influence of extreme pixel points. The extracted radiomic features were normalized to a standard unit using the following equation: f(x) = 1000 ∗ (χ − µ_χ_)/σ_χ_, where µ_χ_ and σ_χ_ denote the mean and standard deviation of the image intensity, respectively. Expounded description regarding the initial settings used in FeAture Explorer Pro for the feature extraction process are provided in Supplementary Appendix 1. Image types includes original image and extract feature categories. Eventually, of 107 radiomics features extracted from the 3D-dimensional region were composed of three types, that is, shape features (number of features [m] = 18), first-order features (m = 14) and texture features (m = 75). Texture features include 24 Gy level co-occurrence matrix (GLCM) features, 16 Gy level run length matrix (GLRLM) features, 16 Gy level size zone matrix (GLSZM) features, 5 neighborhood gray tone difference matrix (NGTDM) features, 14 Gy level dependency matrix (GLDM) features. The intra-class correlation coefficients (ICCs) of the features were calculated to evaluate the inter-observer reproducibility and the features with ICC > 0.80 were enrolled in subsequent analysis. The derivation and internal validation sets are split in a ratio of 7:3. We up-samples by repeating random cases to balance the samples of micropapillary and solid negative and positive. The L2 norm was computed and divided by each feature vector. The feature vector was then mapped into a unit vector. We examined the similarity of each feature pair and eliminated one if its PCC (Pearson Correlation Coefficient) value which was greater than 0.99 to reduce the dimension of the feature space. We used recursive feature elimination (RFE) to select radiomics features based on a classifier by repeatedly considering a smaller set of features. Analysis of variance (ANOVA) was used to investigate the significant features associated with the labels. We sorted features according to their corresponding feature value (F-value), which were calculated to determine the relationship between features and labels, and selected a specific number of features to build the optimal integrated model. To identify predictive features in the model, we used logistic regression with the LASSO (Least absolute shrinkage and selection operator). The final lost function was augmented with the L1 norm, and the weights were constrained. The radiomics models′ hyper-parameters were based on the model’s performance on the internal validation data set. Figure S1A–E depicts the automated segmentation process, features′ reproducibility analysis, feature selection and model development.

The radiomics model derived from the Least absolute shrinkage and selection operator (LASSO) procedure using fivefold cross validation in the derivation set without wavelet features.

### Deep learning network architecture implementation

**Figure 2:**
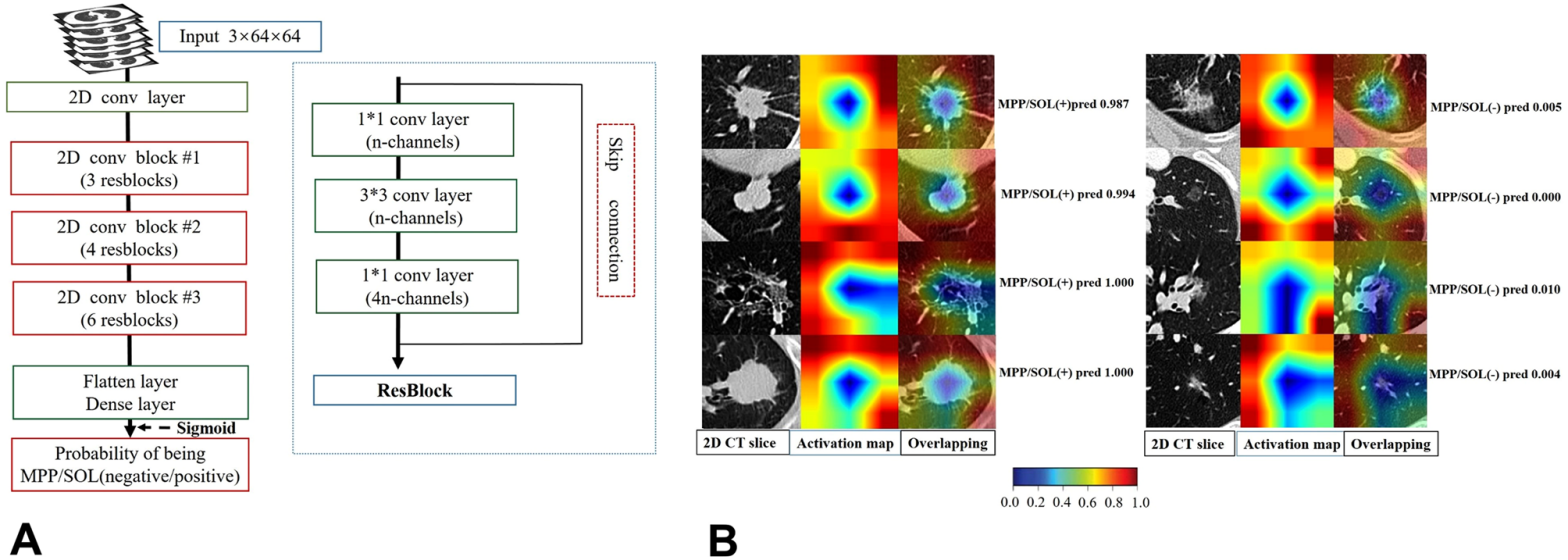
The architecture of WRN network and the prediction diagnosis of DL-score for the MPP/SOL positive lesion. (**A**) The illustrated WRN50 architecture was presented in our study which is conducted by PyTorch (version 1.6.0; http://pytorch.org). (**B**) From the left, the first column represents 2.5D patches with a size of 3 × 3 × 64 pixels that is locally exhibited the tumor and the second column reveals the activation heat maps. Last column makes a good visual reference for the prediction probability of MPP/SOL negative/positive, that is DL-score, which is observed by the importance of each part of tumor generated by ("Strix" (https://github.com/Project-Strix/Strix)). *WRN* wide residual network; *DL-score* deep learning score**.**

In this study, the deep learning model was developed using a derivation set based on the “wide residual network” (WRN) architecture^22^, The architecture of WRN50 is consisted of 50 convolutional layers with 4 max-pooling layers. We fed 2.5D patches with a size of 3 × 3 × 64 pixels into the neural network. Each patch was cropped to contain the maximum area of the nodule's slice. Three adjacent slices were extracted and concatenated to form a 3-channel patch (Fig. 2A). The chest images were used as input data, deep learning score (DL-score) is the output of the network which was transferred by sigmoid function as the probability of being MPP/SOL negative or MPP/SOL positive subtype. All annotated CT images were used as input material to create a heat map illustrating a sensitive region of interest (ROI), which showed the regions with the greatest impact on the final prediction layer of the input images^23^. We used data augmentation techniques to suppress the problem of overfitting in derivation phase. Stochastic gradient descent (SGD) optimizer was employed to train the network with binary cross-entropy loss. Five-cross validation was used to evaluate the quality of proposed model and avoid over fitting and under fitting. PyTorch (version 1.6.0; https://pytorch.org) was used to implement the algorithm. A gradient-weighted activation mapping (Grad-CAM)^15,23^ is a commonly used method in computer vision field to provide interpretability. In this work, we used Grad-CAM to visualize the important regions of the input image data (Fig. 2B). Higher value indicated the more indicative CT areas for associated prediction-making. The Grad-CAM algorithm was implemented by our deep learning framework "Strix" (https://github.com/Project-Strix/Strix) constructed in PyTorch (version 1.6.0; https://pytorch.org). The output of the final dense layer was our deep learning signature, which was transferred with the sigmoid function as the DL-score and built DL model.

### Combined model built

Logistic regression analysis was conducted to develop the model 4 with the incorporation of the selected meaningful clinical features, DL-score and R-score to construct the combined model on the derivation set.

**Figure 3:**
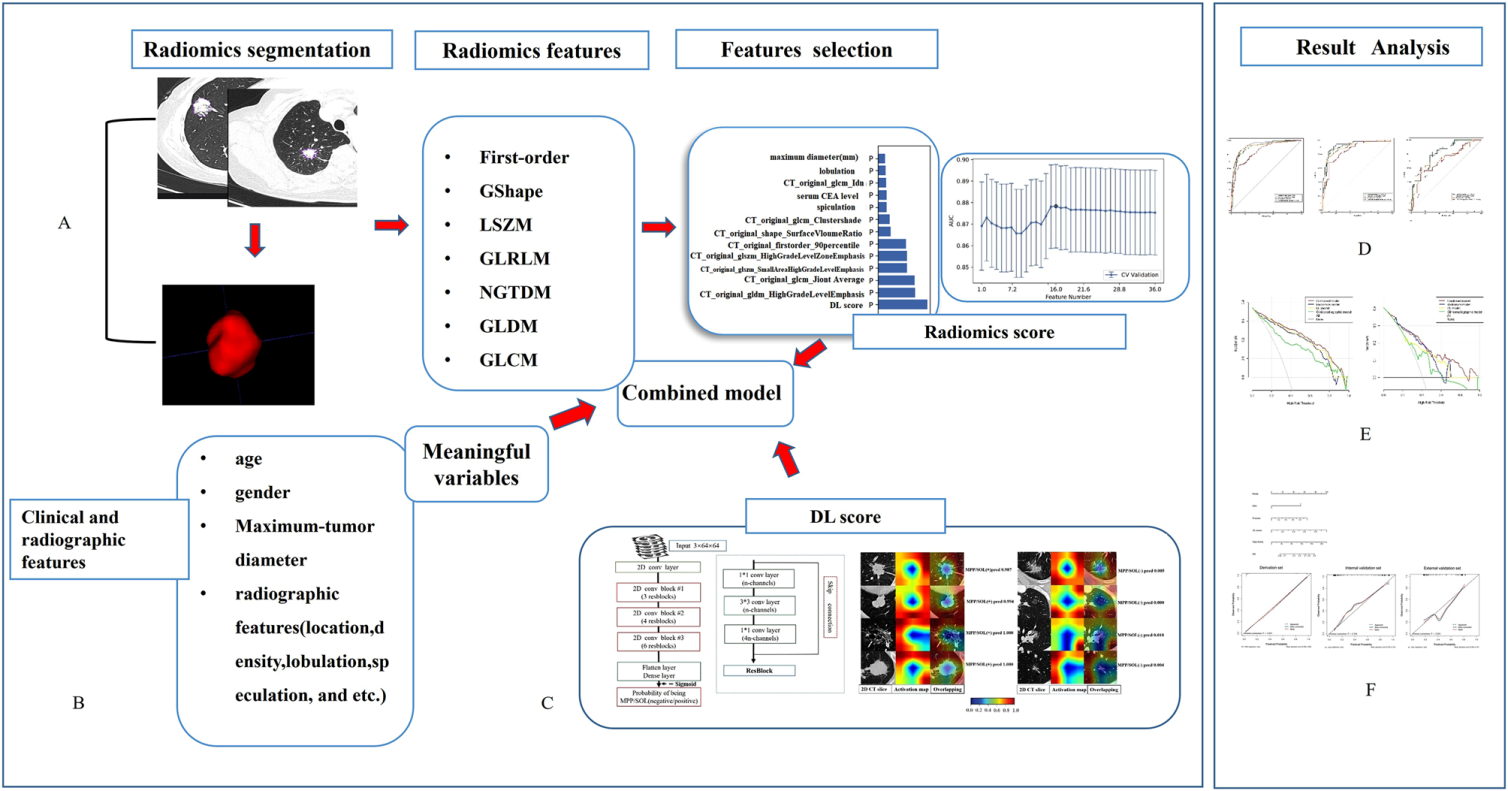
Workflow of the development of different models. Left: Top row; (**A**) The step of radiomics analysis includes segmentation of CT images, feature extraction and selection by FeAture Explorer Pro (FAE Pro, Version 0.3.7, (https://github.com/salan668/FAE.git). Bottom row; (**B**)The clinicoradiographic model development. (**C**) Deep learning model was developed by PyTorch (version 1.6.0; http://pytorch.org) and DL -score generated from ("Strix" (https://github.com/Project-Strix/Strix)). Right: Top row; (**D**) The ROC comparison between different models. Middle row; (**E**) The DCA curve plotted by rmda package in R software (The R Founding: http://www.r-project.org; Version 4.1.2); Bottom row; (**F**) The nomogram and calibration analysis executed by rms package in R software, ResourceSelection package for Hosmer–Lemeshow test.

Figure 3 illustrates the steps involved in our study, including clinicoradiographic features selection, segmentation of CT images, feature extraction and selection from radiomics, deep learning network construction, combined model construction and model analysis.

### Clinical utility and prediction nomogram

We internally assessed the models' performance in independent internal validation data using the receiver operating characteristic (ROC) curve analysis. The prediction nomogram was plotted based on logistic regression based on the combined model. Consistency between the nomogram was implemented with Hosmer–Lemeshow goodness-of-fit (GOF) test using calibration curves via bootstrapping with 1000 resamples^24^. The clinical usefulness was estimated with decision curve analysis and with the visualization of decision curve and clinical impact curve^25^.

### Statistical analysis

Statistical analyses were carried out using IBM SPSS software (version 22.0, http://www.ibm.com), R software (The R Founding: http://www.r-project.org; Version 4.1.2), MedCalc (version 15.0, Mariakerke, Belgium), PyTorch (version 1.6.0; https://pytorch.org) and FeAture Explorer Pro (FAE Pro, V0. 3.7) on Python (3.7.6). The rms package in R software was implemented for calibration analysis, ResourceSelection package for Hosmer–Lemeshow test, rmda for DCA curve analysis. PyTorch (version 1.6.0; https://pytorch.org) was adopted to construct the deep learning model and Grad-CAM. The characteristics were compared using the Student t test or Mann–Whitney U test (for continuous parameters expressed as mean ± standard deviation or median ± interquartile range) and Chi-square or Fisher's exact tests or (for categorical variables expressed as numbers). The criteria of intra-class correlation coefficient (ICC) value are graded as reported by the precious study^26^: 0 ~ 0.20, slight; 0.21 ~ 0.40, fair; 0.41 ~ 0.60, moderate; 0.61 ~ 0.80, substantial; 0.81 ~ 1.00, almost perfect agreement. Five-fold cross validation was employed to estimate the performance of the radiomics and deep learning model in the training procedure. Moreover, external validation set was performed for these models′ validation. The AUC comparison was processed with DeLong′ s test.

## Results

Supplementary Table S1 presents that there were no statistically significant differences in clinic-radiographic characteristics between the derivation and internal validation sets (P all > 0.05).

### Model-development

Table 1 shows the contribution of clinicoradiographic, radiomics, and DL-score features along with their associated coefficients in different models.

**Table 1:** The coefficients of features of four models.

Models	Features coefficient in model	Coefficients	P value	intercept
Model 4	Combination			− 4.173
	R-score	2.405	0.0134	
	CEA (≥ 4.70 ng/ml)	1.966	0.0002	
	DL-score	3.722	< 0.001	
Model 3	DL-score	–	< 0.001	−
Model 2	Radiomics signature			− 0.057
	CT_original_firstorder_90Percentile	0.989	< 0.001	
	CT_original_firstorder_Mean	3.881	< 0.001	
	CT_original_gldm_SmallDependenceHighGrayLevelEmphasis	0.261	< 0.001	
	CT_original_glszm_SizeZoneNonUniformityNormalized	2.974	< 0.001	
	CT_original_shape_SurfaceVolumeRatio	− 3.245	< 0.001	
Model 1	Clincoradiographic feature			1.900
	CEA (≥ 4.70 ng/ml)	− 1.897	< 0.001	
	Maximum-tumor diameter	0.053	0.012	
	Lobulation	−1.589	< 0.001	
	Spiculation	− 1.378	< 0.001	

*CEA* carcinoembryonic antigen.

#### Model 1: clinicoradiographic features

The consistency analysis of radiographic variables between two radiologists are listed in Table 2. Comparison of clinical and radiographic features between MPP/SOL positive and MPP/SOL negative in derivation and internal validation set are presented in Tables 3 and 4. The finally selected features with logistic regression in derivation set revealed that the independent predictor for MPP/SOL positive lesions included serum CEA level, maximum-tumor diameter, lobulation and spiculation. The calculation formula was as follows: (− 1.897) * CEA (≥ 4.70 ng/mL) + 0.053*maximum-tumor diameter + (− 1.589) * lobulation + (− 1.378) * spiculation + 1.900.

**Table 2:** The consistency analysis of radiographic variables between two radiologists.

Variables	ICC (95%CI)	Kappa values(95%CI)
Maximum-tumor diameter(:mm)	0.928 (0.915 ~ 0.940)	
Lesion type		0.880 ± 0.021(0.840 ~ 0.921)
Spiculation		0.755 ± 0.033(0.690 ~ 0.820)
Lobulation		0.722 ± 0.029(0.665 ~ 0.778)
Bubble-like appearance		0.872 ± 0.036(0.801 ~ 0.943)
Lung-tumor interface		0.792 ± 0.031(0.731 ~ 0.852)
Change in vessels		0.769 ± 0.032(0.706 ~ 0.832)
Pleural tag and indentation		0.890 ± 0.013(0.864 ~ 0.916)
Tumor-bronchus relation		0.880 ± 0.022(0.864 ~ 0.930)
Cavity		0.703 ± 0.081(0.544 ~ 0.862)

*ICC* intraclass correlation coefficient.

**Table3:** Comparison of clinical features between MPP/SOL positive and MPP/SOL negative.

Features	Derivation set (134 /226)	*p* value	Internal validation set (57/97)	*p* value
	No. of LUNG ADC with MPP/SOL+/−		No. of LUNG ADC with MPP/SOL+/−	
Tumor location				
RUL	41(30.6)/62(27.4)	0.151	16(28.1)/27(27.8)	0.119
RML	28(20.9)/27(12.0)		3(5.3)/18(18.6)	
RLL	33(24.6)/76(33.6)		22(38.6)/38(39.2)	
LUL	10(7.5)/18(8.0)		7(12.3)/5(5.2)	
LLL	22(16.4)/43(19.0)		9(15.8)/9(9.3)	
Age	61.0 ± 9.4/58.3 ± 10.6	0.016	61.0 ± 10.0/58.6 ± 9.9	0.139
Male	77(57.5)/85(37.6)	< 0.001	33(57.9)/33(34.0)	0.004
Family history	5(3.7)/6(2.7)	0.566	1(17.5)/2(2.1)	1.000
Smoking history	100(74.6)/28(12.4)	0.002	18(31.6)/12(12.4)	0.004
Serum CEA value	49(36.6)/10(4.4)	< 0.001	18(31.6)/4(4.1)	< 0.001
Serum CYFRA21-1 value	25(18.7)/21(9.3)	0.010	9(47.4)/16(59.8)	0.09
Serum NSE value	69(51.5)/109(48.2)	0.550	27(47.4)/58(59.8)	0.134
CEA level(ng/ml)	3.2(1.9,6.2)/1.8(1.3,2.7)	< 0.001	1.9 (1.2, 2.6)/2.7(1.9,5.6)	< 0.001
NSE level(ng/ml)	16.5(13.9,21.1)/16.0(13. 5,20.4)	0.233	17.1(15.0,18.8)/16.1(13.9,19.9)	0.288
CYFRA211level(ng/ml)	2.5(1.8,3.0)/2.0(1.6,2.5)	< 0.001	2.0(1.6,2.7)/2.3(1.8,3.0)	0.135

Data are numbers of patients and parentheses indicate the proportion.

*CEA* carcinoembryonic antigen, *NSE* neuro-specific enolase, *CYFRA21-1* cytokeratin fragment 21–1.

**Table 4:** Comparison of radiographic features between MPP/SOL positive and MPP/SOL negative.

Features	Derivation set (134/226)	*p* value	Internal validation set (57/97)	*p* value
	No. of LUNG ADC with MPP/SOL+/−		No. of LUNG ADC with MPP/SOL+/−	
Maximum-tumor diameter(:mm)	16.32(11.30,22.23)/10.69(8.50,14.70)	< 0.001	12.95(15.4,20.25)/11.10(8.25,13.65)	< 0.001
C/T ratio	0.44(0.81,1.00)/0.87(0.34,1.00)	0.847	0.90(0.38,1.00)/0.88(0.46,1.00)	0.905
Lesion type				
pGG	22(16.4)/27(11.9)		1(1.8)/17(17.5)	
mGG	63(47.0)/103(45.6)	0.367	7(12.3)/51(52.6)	< 0.001
Solid	49(36.6)/96(42.5)		49(86.0)/29(29.9)	
Lung-tumor interface (blur)	106(79.1)/96(42.5)	0.001	16(28.1)/21(21.7)	0.369
Spiculation (present)	72(53.7)/30(13.3)	< 0.001	32(56.1)/12(12.4)	< 0.001
Lobulation (present)	106(79.1)/96(42.5)	0.001	45(79.0)/43(44.3)	< 0.001
Cavity (present)	15(7.6)/12(5.3)	0.025	2(3.5)/1(1.0)	0.284
Bubble-like appearance (present)	4(3.0)/11(4.9)	0.038	9(15.8)/7(7.2)	0.093
Change in vessels (present)	42(31.3)/66(29.2)	0.699	15(26.3)/28(28.9)	0.734
Tumor-bronchus relation(present)	75(56.0)/54(23.89)	< 0.001	35(61.40)/22(22.7)	< 0.001
Pleural tag and indentation (present)	86(64.2)/70(31.0)	< 0.001	35(61.4)/24(24.7)	0.734
Predominant histologic subtypes		< 0.001		
Lepidic	5(3.7)/76(33.9)		4(7.0)/35(36.1)	
Acinar	38(28.4)/102(45.5)		9(15.8)/44(45.4)	
Papillary	1(0.7)/6(2.7)		0(0)/3(3.0)	< 0.001
Micropapillary	17(12.7)/0(0)		7(12.3)/0(0)	
Solid	44(32.8)/0(0)		25(43.9)/0(0)	
Mixed type	29(21.6)/42(18.8)		12(21.0)/15(15.5)	

Data are numbers of patients and parentheses indicate the proportion.

*C/Tratio* consolidation/tumor diameter ratio, *pGG* pure Ground Glass density, *mGG* mixed Ground Glass density.

#### Model 2: Radiomics model

The 107 features whose ICC value in intra-observer (0.802 ~ 0.999) and inter-observer (0.809 ~ 0.999) were included in the subsequent analysis. Feature selection includes 16 radiomics features (Fig. S1C) and eventually, 5 non-zero coefficient features (Fig. S1D) were remained. Radiomics score was calculated by the following formula:

(R-score) = 0.989*CT_original_firstorder_90Percentile + 3.881* CT_original_firstorder_Mean + 0.261* CT_original_gldm_SmallDependenceHighGrayLevelEmphasis + 2.974 * CT_original_glszm_SizeZoneNonUniformityNormalized + (-3.245) * CT_original_shape_SurfaceVolumeRatio + (− 0.0570).

**Figure 4:**
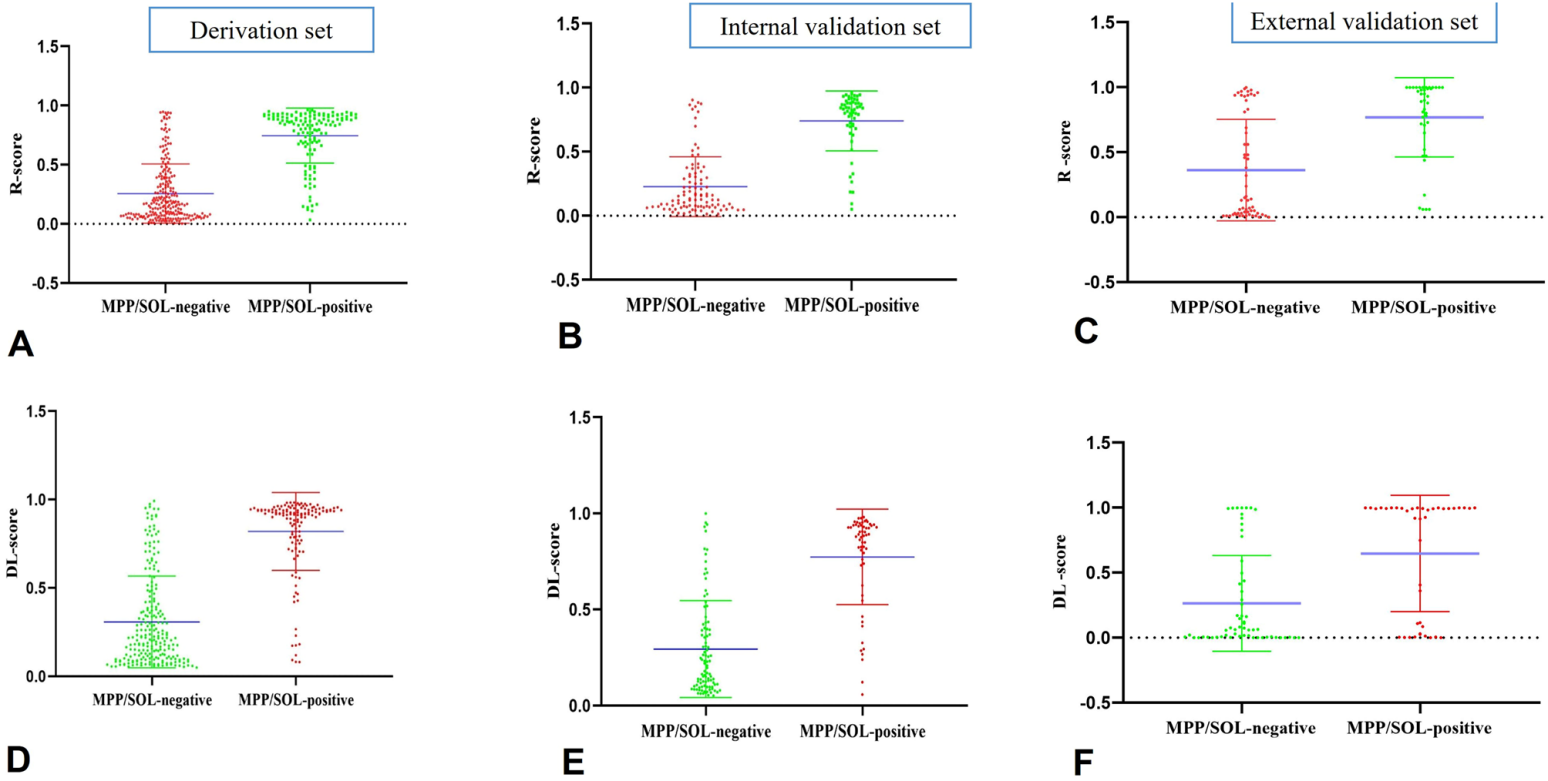
Dot diagram of R-score and DL-score for differentiating the lung lesion with MPP/SOL positive. Dot diagrams show that the value of the R-score is significantly higher in patients of lung ADC with MPP/SOL positive in the derivation set (**A**), the Internal validation set (**B**) and external validation set (**C**) (p all < 0.001). The value of DL-score is also obviously higher in patients of lung ADC with MPP/SOL positive in the derivation set (**D**), the Internal validation set (**E**) and external validation set (**F**) (p all < 0.001). R-score = radiomics score; DL-score = deep learning score.

The R-score existed significant differences between the MPP/SOL + and MPP/SOL− groups in the derivation set (0.164(0.707,0.382) vs 0.846(0.681,0.913), p < 0.001) and the same result in the internal validation set (0.127(0.070,0.306) vs 0.829(0.700,0.884), p < 0.001) and external validation set (0.135(0.020,0.825) vs 0.900(0.680,1.000) (Fig. 4A–C). The cut-off value of R-score is 0.582 chosen to maximize the Youden index of the ROC analysis from the derivation set.

#### Model 3: Deep Learning model

The model was constructed based on the DL-score. Both the DL-score of derivation set (0.216(0.102,0.421) vs 0.918(0.781,0.942), p = 0.001) (Fig. 4A,B, p < 0.001) and that of internal validation set ((0.197(0.100,0.400) vs 0.888(0.735,0.938), p < 0.001), and external validation set ((0.063(0.003,0.432) vs 0.98(0.058,0.998), p < 0.001) showed significant statistical significance in DL-score between the MPP/SOL+ and MPP/SOL− groups. (Fig. 4D–F). The cut-off value from derivation set is 0.656 chosen to maximize the Youden index of the ROC analysis.

#### Model 4: combined model

The features′ dimensionality was reduced from 107 to 16 features. And non-zero coefficients of the finally calculated as R-score combined with clinicoradiologic variables and DL-score in derivation set revealed that serum CEA (≥ 4.70 ng/ml), DL-score and R-score were independent predictor for MPP/SOL positive lesions based on logistic regression. The calculation formula = 3.722*DL-score + 2.405*R-score + 1.966*serum CEA level (≥ 4.70 ng/ml) + (− 4.173).

### Performance and comparison of the different models

**Figure 5:**
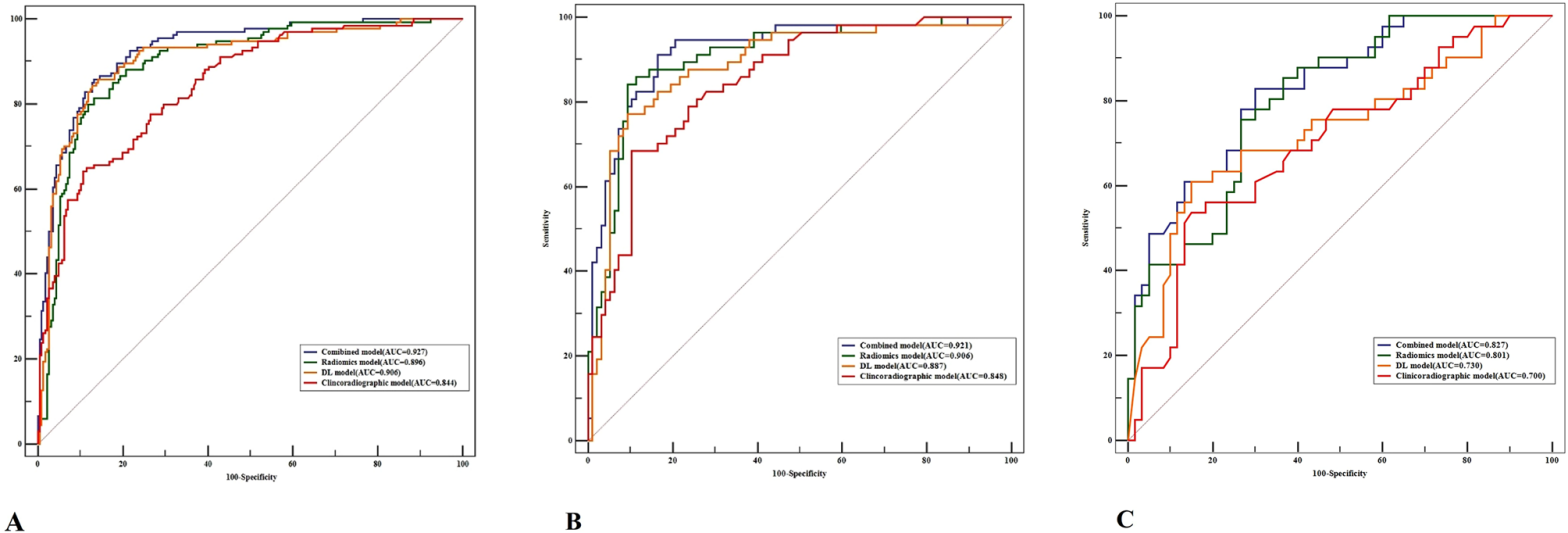
The ROC curve and AUC comparison of five different models. left to right: the ROC curve and AUC comparison of in derivation (**A**), Internal validation dataset (**B**) and external validation (**C**), respectively. The ROC curve in three datasets showed the trend that the DL-score based and radiomics feature based scheme can improve the clinicoradiographic model's performance. Comparison between the DL-score based and radiomics feature based scheme (model 3 and model 2) exhibited the DL model is only superior to radiomics model in derivation set (0.906 vs 0.896). In a comparison with the combined scheme model 4 yielded moderately higher performance (AUC = 0.929 in the derivation set, AUC = 0.921 in Internal validation set and AUC = 0.827 in the external validation set, respectively) than model 3 and model 1.

Table 5 shows the AUC (95%CI), accuracy, sensitivity, specificity, PPV and NPV of different models in the derivation, internal validation and external validation sets. The ROC curve and AUC values comparison associated with the derivation, internal validation and external validation set are shown in Fig. 5A–C. In the internal validation dataset, Table 5 demonstrated that the model 4 based on 3 features had the maximum AUC. The AUC and accuracy could be as high as 0.921 (95% CI 0.866 ~ 0.958) and 84.7%, respectively. Internally, the combined model (model 4) demonstrated a sensitivity of 91.2%; specificity of 83.5%; a PPV of 76.5%; and an NPV of 94.2%. And the external validation achieved a sensitivity of 83.9%, specificity of 70.0%; a PPV of 65.4%; and an NPV of 85.7%. The clinicoradiograhic model for classifying MPP/SOL negative vs. positive obtained an accuracy of 80.9% and 72.3% in internal and external validation set. The radiomics model and DL models performed with an accuracy of 88.3% and 85.7%, respectively. We had performed DeLong's test to analyze the difference between the AUCs of different models in the internal and external validation and derivation datasets, respectively. Table 6 showed merely significant difference between model 4 and model 3 (p = 0.022), model 4 and model 1(p = 0.010) in the internal validation set. Externally, the diagnostic performance of the combined model (0.827, 95% CI 0.739–0.895) was higher than those of radiomics model (0.801, 95% CI 0.710–0.874) (P = 0.047), DL model (0.730, 95% CI 0.633–0.814) (P = 0.036) and clinicoradiographic model (0.700, 95%CI 0.601–0.787) (P = 0.016). The DL model showed comparable predictive performance to the radiomics model internally and externally. Note however, the AUCs of radiomics and DL models are superior to that of clinicoradiographic model, but there were no significantly statistical differences between models 3 and model 1 (AUC: 0.887 vs 0.848, p = 0.257) in internal validation set and between model 2 and model 1 (AUC: 0.906 vs 0.848, p = 0.063), which concomitantly occurred in the externally validation((P = 0.074, P = 0.649, respectively). Even the integrated model 4 achieved higher AUC (0.929, 95%CI:0.897 ~ 0.954) in the derivation set and AUC (0.921, 95% CI 0.866 ~ 0.958) in the internal validation set than the other 3 models, resulting in a halfway respectable outcome when compared to model 2 (AUC 0.921 vs 0.906, P = 0.360) internally, when compared externally to model 1 and model 3, the model 4 (AUC 0.827,95%CI:0.739-0.895) also obtained the same diagnostic efficacy. Additionally, the AUCs of 5-cross validation in radiomics and deep learning training procedure were 0.907 (95% CI 0.8938–0.9223) and 0.899 (95%CI 0.859–0.918), respectively.

**Table 5:** The diagnosis of different types of models for lung ADC with MPP/SOL pattern.

Models		AUC	95%CI	SEN (%)	SPE (%)	ACC (%)	NPV (%)	PPV (%)
Model 1	Derivation set	0.844	0.803–0.880	64.2	89.4	80.0	80.8	78.2
	Internal validation set	0.848	0.806–0.919	75.4	86.6	80.9	85.7	76.8
	External validation set	0.700	0.601–0.787	53.66	85.0	72.3	72.9	71.0
Model 2	Derivation set	0.896	0.861–0.930	81.3	86.7	84.7	88.7	78.4
	Internal validation set	0.906	0.848–0.947	84.2	90.7	88.3	90.7	84.2
	External validation set	0.801	0.710–0.874	75.6	73.3	74.3	81.5	75.6
Model 3	Derivation set	0.906	0.865–0.930	85.1	85.4	85.7	92.5	73.1
	Internal validation set	0.887	0.826–0.932	77.2	90.7	84.1	87.1	80.0
	External validation set	0.730	0.633–0.814	61.0	85.0	75.2	76.1	73.5
Model 4	Derivation set	0.929	0.898–0.954	85.8	86.7	86.1	90.7	79.2
	Internal validation set	0.921	0.866–0.958	91.2	83.5	84.7	94.2	76.5
	External validation set	0.827	0.739–0.895	83.9	70.0	75.2	85.7	65.4

*AUC* area under curve, *CI* confidence interval, *NPV* negative predictive value, *PPV* positive predictive value, *SEN* sensitivity, *SPE* specificity, *ACC* accuracy.

**Table 6:** Comparison and evaluation the performance of different models in the three datasets.

Models (AUC)	Derivation set (360)	*p value*	Internal validation set (154)	*p value*	External validation set (101)	*p value*
Model 2 vs. Model 1	0.896 vs 0.844	**0.014**	0.906 vs 0.848	0.186	0.801 vs 0.700	0.074
Model 3 vs. Model 1	0.906 vs 0.844	**0.012**	0.887 vs 0.848	0.257	0.730 vs 0.700	0.649
Model 4 vs. Model 1	0.929 vs 0.844	< **0.001**	0.921vs 0.848	**0.009**	0.827 vs 0.700	**0.016**
Model 3 vs. Model 2	0.906 vs 0.896	0.496	0.887 vs 0.906	0.320	0.730 vs 0.801	0.193
Model 4 vs. Model 3	0.929 vs 0.906	0.058	0.921 vs 0.887	**0.016**	0.827 vs 0.730	**0.047**
Model 4 vs. Model 2	0.929 vs 0.896	**0.020**	0.921 vs 0.906	0.360	0.827 vs 0.801	**0.036**

*AUC* area under the curve. Significant values are in bold.

### A prediction nomogram building

**Figure 6:**
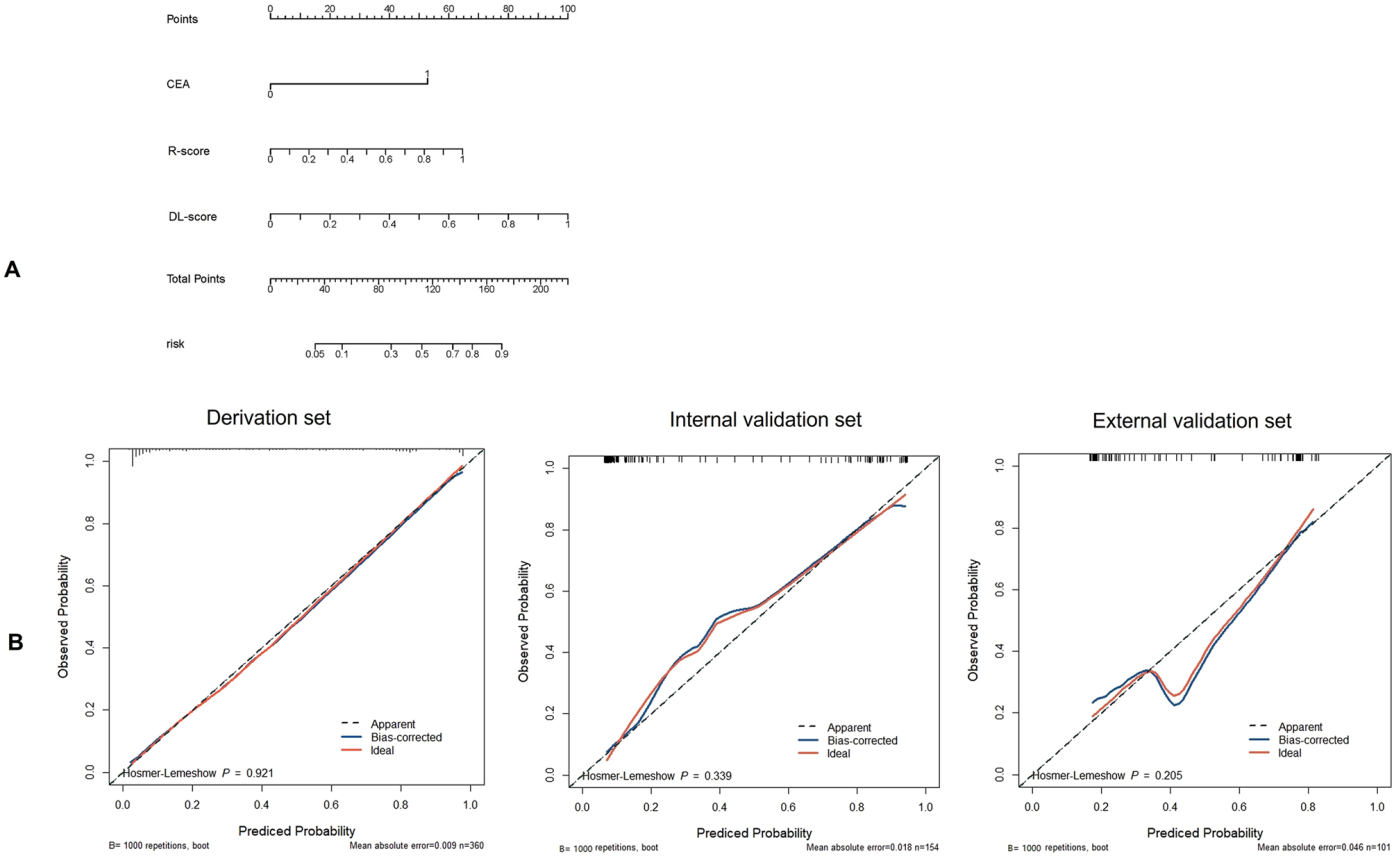
The nomogram and calibrate curve based on combined model. (**A**) The nomogram based on combined model was developed in the derivation set. And the R-score, DL-score and CEA were incorporated by rms package in R software. (**B**) Calibration curves of the combined model indicated good agreement between predicted probability and actual occurrence in derivation set (Hosmer–Lemeshow test, P = 0.921), internal validation set (0.309) and external validation set (P = 0.205).

The prediction nomogram was plotted based on logistic regression on the basis of the combined model, which is added significant incremental performance to the clinical model (Fig. 6A). Favorable calibration of the nomogram corroborated both in the derivation, internal validation set (Fig. 6B) and external validation set (Fig. 6B) demonstrated good calibration with p value of 0.921, 0.339 and 0.205, in the derivation, internal and external validation set in the Hosmer and Lemeshow goodness of fit (GOF) test, respectively.

### Clinical practice

**Figure 7:**
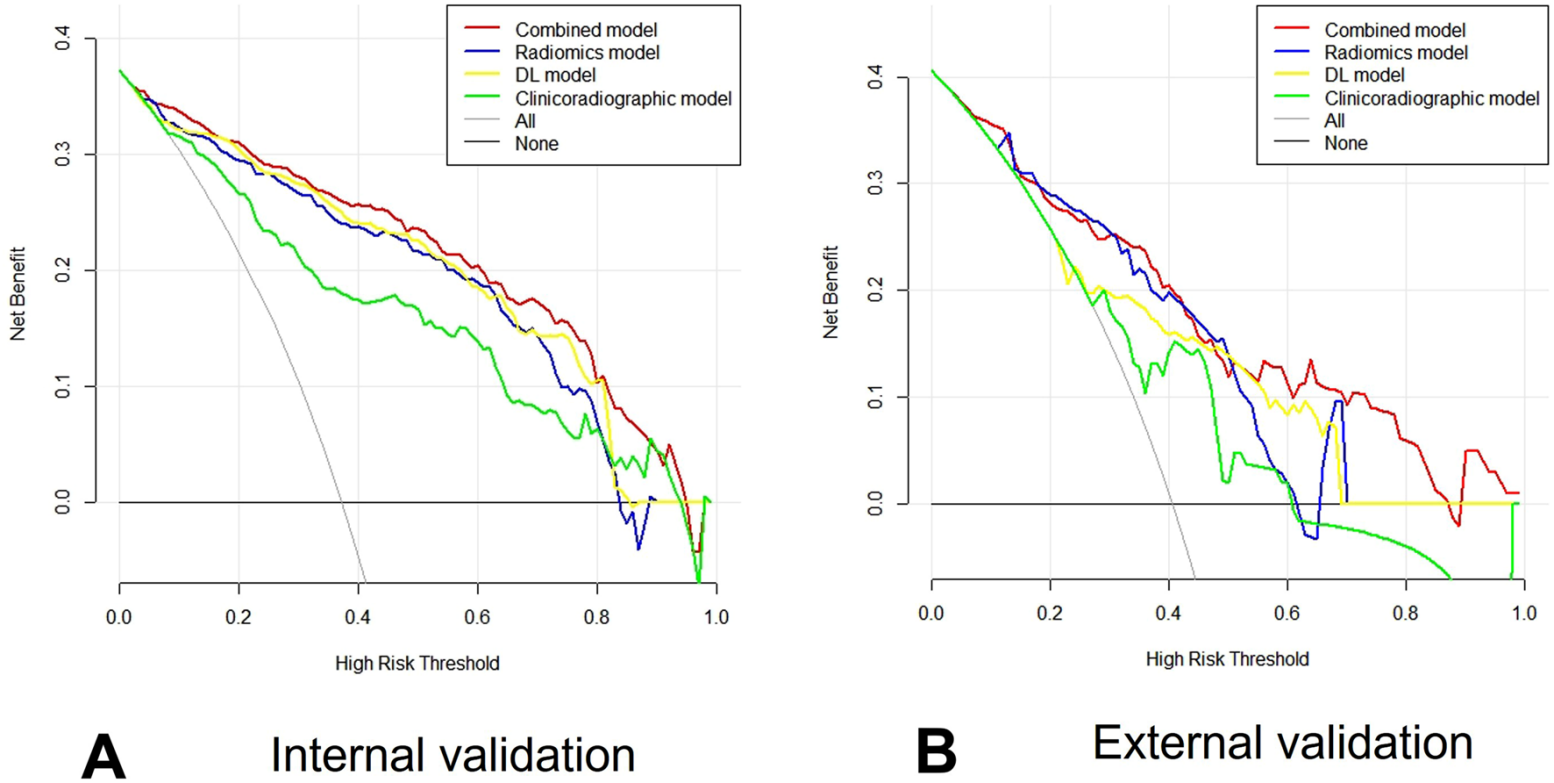
Decision curve analysis for the different models. The DCA for the different models showing the benefit of following prediction models at diverse probability thresholds for adverse events in the internal validation set (**A**) and external validation set (**B**). The y-axis indicates the net benefits and the x-axis represents the different probability thresholds of being MPP/SOL positive. The black line and gray represent the assumption that no patients with MPP/SOL positive and all patients with MPP/SOL positive, respectively.

Decision curve analysis (DCA) revealed that indicating that the radiomics model, DL model or the combined model achieved moderately better net benefits than the clinical and radiographic factors or DL -score alone to predict lung ADC with MPP/SOL positive at threshold probability (5–80%) and comparable better net benefits to the radiomics model in internal validation set (Fig. 7A). If the threshold probability of a patient in a range of (48–52%) and (56–88%), the combined model used to predict positive lesions would be more beneficial than DL model and clinicoradiographic model (Fig. 7B). Clinical impact curve (Fig. S2A,B) shows the estimated number who would be affirmed high risk for each risk threshold and visually shows the proportion of those true positive cases^24^^.^

## Discussion

To date, lots of study has developed invasive preoperative model for assessment of lung ADC with MPP/SOL patterns. Our study extended previous work^7–10,27,28^ by decoding the phenotypes of lung ADC using clinical, quantitative radiomics features and DL-score based scheme model separately and combined, attempting to determine whether an integrated strategy incorporating clinical, radiomics, and DL-score features could be used to discriminate lung ADC with MPP/SOL pattern for a more effective surgical approach or subsequent treatment scheme. The proposed combined model with a sensitivity of 91.2% and 83.9%, accuracy of 84.7%, 75.2%, AUC of 0.921 and 0.827 in internal and external validation, respectively, was significantly superior to deep learning and clinicoradiographic model alone (P < 0.05).

Firstly, we developed a clinicoradiographic model (model 1) to classify lung ADC with or without MPP/SOL pattern using clinical, imaging, and histopathological profiling data from 625 lung ADC (internal dataset: external dataset; 514:101). Morphological CT features, such as lobulation, spiculation and maximum -tumor diameter all contribute to the differential diagnosis of MPP/SOL negative and positive. Our study revealed that the more lobulation, spiculation and bigger maximum-tumor diameter observed in lung ADC, the higher the probability that the lesion is a lung ADC with MPP/SOL pattern, which is comparably consistent with the findings of Park and his colleagues^13^ that solid-predominant adenocarcinomas are more likely to exhibit spiculation or lobulation, which could be explained by enrolment bias. Clinical features such as serum biomarkers are widely believed to be a useful blood-based diagnosis for lung adenocarcinoma^29^, particularly the serum CEA level. The findings indicated that abnormal serum CEA levels are more prevalent in lung ADC with MPP/SOL positive than in MPP/SOL negative, but further study has more samples need to confirm it in future. Several studies^11,15^ focused on measuring the C/T ratio as the main morphological predictor on invasiveness histological lesions in lung ADC, especially in part-solid nodules. The C/T ratio calculated in our study showed no statistical significance in derivation and internal validation set. It may be resulting from the limited various density nodules sample size, even though we attempt to randomly distribute the dataset with the aid of machine operation. Chen et al.^15^ investigated the C/T ratio discernibility of lung ADC with MPP/SOL with AUC of 0.850, which is equal to the detection ability of radiomics model. Regarding the result of performance of C/T ratio, we considered patient selection bias to be the reason, that is the case of lung ADC with MPP/SOL is relatively fewer than another lung ADC pattern mainly featuring part-solid density.

Secondly, we compared to models built using DL model (model 3) and radiomics model (model 2) separately, the model 3 produced equivalent performance (P = 0.320). It was concluded that a DL model was feasible for classifying lung ADC with MPP/SOL pattern. However, the classification performance of the model 3 was inferior to that of model 2 in the validation set (AUC 0.906 vs 0.887), but superior in the derivation set (AUC 0.906 vs 0.896), suggesting that overfitting might occurred during the derivation process^14^^.^ And previous study^30^ pointed that the opaqueness of deep learning results from implicit feature engineering or modeling makes its application a little difficult in clinic practice. In contrast, the radiomics features may be more feasible considering the standardized radiomics process, platform of tumor segmentation and model development^31^.

As we all known, the combination of radiomics features into R-score value can simplify the analysis flow. Finally, we attempted to combine R-score and DL-score with selected clinical findings to establish combined models to determine the predictive power and clinical relevance of lung ADC subtype discrimination. We discovered that model 4 had the best performance in preoperatively diagnosing MPP/SOL pattern in lung ADC patients with an AUC (0.921) in the internal validation set outperforming the model 3 and model 1, however, achieving a halfway decent result compared to model 2. We hypothesized that the radiomics model had sufficient signature for prediction and that additional variables could not significantly improve the discriminating capacity. The model 2 obtained a high level of accuracy (88.3%, 84.7%) in internal validation and derivation datasets, slightly higher than the model 3 (84.1%, 85.7%) and model 4 (84.7%, 86.1%). However, when compared externally, the mode 4 yielded the same accuracy with the model 3 (75.2%), which is little higher than model 2 (74.3%). The most likely explanation for this phenomenon is insufficient samples and human intervention during the deep learning network derivation process^29^. The combined model is superior to another 3 model alone in external validation set (P all < 0.05). We hypothesized that the result indirectly reflected the classification performance of the DL model and associated fusion model in lung ADC, which may be affected by the overfitting problem during deep learning model’s data processing. Our study demonstrates that combining CT-based radiomics and DL-score with clinical implications would be another method for preoperative diagnosis of lung ADC with MPP/SOL pattern, but it may not be prudent to combine R-score, DL-score and clinical features. Therefore, further research and development of novel fusion methods for fusing various types of features will need to be conducted.

As regards the issue of batch effect due to the utility of different CT devices, reported in some study^33,34^, we benefit form Qu et al.^34^ study, firstly adressing the principal component analysis (PCA) on the selected radiomics features to detect the batch effect. The results elucidated that no significant batch effects ware observed among the data obtained by different CT scanners (shown in Fig. S3). As a result, statistical harmonization methods such as ComBat was not subsequently implemented to calibrate the data.

This study has the following limitations. First, the opaque nature of deep learning mechanism may be a source of contention^28^, as does the information-fusion strategy. Second, we did not pay enough attention in our investigation to the component of MPP and SOL structure, and lung ADC with multiple subtypes may be possess typical information of hybrid subtypes rather than that of certain subtypes, thus limiting the subtype discriminability^15^ and producing some choice bias. Although we examined the models' performance internally and externally, the performance of the clinicoradiographic model, radiomics model and DL model is similar with respect to the prediction of the lung ADC with MPP/SOL pattern, a larger prospective dataset may be urgent to affirm these models' performance^30^. Additional samples and data processing ways such as mixup, cutout, and cutmix are required to confirm the impact of deep learning on lung ADC subtypes detection^32^. Finally, human-craft features, such as segmentation reproducible, manual outlining determination accuracy^35^, results floating with the variation of the initial segmentation. Therefore, additional work is required, including the collection of more representative data, searching for more feasible image visualization platform, attempt to other algorithm for image segmentation and more features such as wavelet features need to be further discussed.

## Conclusion

In summary, we have established a combined model based on the DL-score, R-score and clinicoradiographic features to distinguish lung ADC with MPP/SOL structure. In comparison to the clinicoradiographic, radiomics features and DL-score-based scheme model separately, the new combined model enhanced the classification performance of clinicoradiologic features and deep learning model. So, the combined model may assist radiologists in differentiating lung ADC with MPP/SOL pattern. The results are still warranted to be certified in more further studies.

## Supplementary Information

Supplementary Information.

## Data Availability

The datasets used and/or analyzed during the current study are available from the corresponding author on reasonable request.

## References

[1] Sung H, Ferlay J, Siegel RL (2021). Global cancer statistics 2020: GLOBOCAN estimates of incidence and mortality worldwide for 36 cancers in 185 countries. CA Cancer J. Clin..

[2] Zhao Y, Wang R, Shen X (2016). Minor components of micropapillary and solid subtypes in lung adenocarcinoma are predictors of lymph node metastasis and poor prognosis. Ann. Surg. Oncol..

[3] Cha MJ, Lee HY, Lee KS (2014). Micropapillary and solid subtypes of invasive lung adenocarcinoma: Clinical predictors of histopathology and outcome. J. Thorac. Cardiovasc. Surg..

[4] Butnor KJ (2020). Controversies and challenges in the histologic subtyping of lung adenocarcinoma. Transl. Lung Cancer Res..

[5] Yanagawa N, Shiono S, Abiko M (2016). The clinical impact of solid and micropapillary patterns in resected lung adenocarcinoma. J. Thorac. Oncol..

[6] Zhao ZR, Lau RWH, Long H (2018). Novel method for rapid identification of micropapillary or solid components in early-stage lung adenocarcinoma. J. Thorac. Cardiovasc. Surg..

[7] Tsai PC, Yeh YC, Hsu PK (2020). CT-guided core biopsy for peripheral sub-solid pulmonary nodules to predict predominant histological and aggressive subtypes of lung adenocarcinoma. Ann. Surg. Oncol..

[8] Song SH, Park H, Lee G (2017). Imaging phenotyping using radiomics to predict micropapillary pattern within lung adenocarcinoma. J. Thorac. Oncol..

[9] Kim H, Goo JM, Lee KH (2020). Preoperative CT-based deep learning model for predicting disease-free survival in patients with lung adenocarcinomas. Radiology.

[10] Wang X, Zhang L, Yang X (2020). Deep learning combined with radiomics may optimize the prediction in differentiating high-grade lung adenocarcinomas in ground glass opacity lesions on CT scans. Eur. J. Radiol..

[11] Chen LW, Yang SM, Wang HJ (2021). Prediction of micropapillary and solid pattern in lung adenocarcinoma using radiomic values extracted from near-pure histopathological subtypes. Eur. Radiol..

[12] He B, Song Y, Wang L (2021). A machine learning-based prediction of the micropapillary/solid growth pattern in invasive lung adenocarcinoma with radiomics. Transl. Lung Cancer Res..

[13] Park S, Lee SM, Noh HN (2020). Differentiation of predominant subtypes of lung adenocarcinoma using a quantitative radiomics approach on CT. Eur. Radiol..

[14] Gao Y, Ding Y, Xiao W (2022). A semi-supervised learning framework for micropapillary adenocarcinoma detection. Int. J. Comput. Assist. Radiol. Surg..

[15] Chen LW, Yang SM, Chuang CC (2022). Solid attenuation components attention deep learning model to predict micropapillary and solid patterns in lung adenocarcinomas on computed tomography. Ann. Surg. Oncol..

[16] Xia X, Gong J, Hao W (2020). Comparison and fusion of deep learning and radiomics features of ground-glass nodules to predict the invasiveness risk of stage-I lung adenocarcinomas in CT scan. Front. Oncol..

[17] Hirsch FR, Franklin WA, Gazdar AF (2001). Early detection of lung cancer: Clinical perspectives of recent advances in biology and radiology. Clin. Cancer Res..

[18] Travis WD, Brambilla E, Noguchi M (2011). International Association for the Study of Lung Cancer/American Thoracic Society/European Respiratory Society: International multidisciplinary classification of lung adenocarcinoma: Executive summary. Proc. Am. Thorac. Soc..

[19] Qi LP, Li XT, Yang Y (2016). Multivariate analysis of pleural invasion of peripheral non-small cell lung cancer-based computed tomography features. J. Comput. Assist. Tomogr..

[20] Qiang JW, Zhou KR, Lu G (2004). The relationship between solitary pulmonary nodules and bronchi: Multi-slice CT-pathological correlation. Clin. Radiol..

[21] Song Y, Zhang J, Zhang YD (2020). FeAture Explorer (FAE): A tool for developing and comparing radiomics models. PLoS One.

[22] Zagoruyko, S. & Komodakis, N. *British Machine Vision Conference, BMVC, September 19–22* (2016).

[23] Hosny A, Parmar C, Coroller TP (2018). Deep learning for lung cancer prognostication: A retrospective multi-cohort radiomics study. PLoS Med..

[24] Kramer AA, Zimmerman JE (2007). Assessing the calibration of mortality benchmarks in critical care: The Hosmer-Lemeshow test revisited. Crit. Care Med..

[25] Kerr KF, Brown MD, Zhu K (2016). Assessing the clinical impact of risk prediction models with decision curves: Guidance for correct interpretation and appropriate use. J. Clin. Oncol..

[26] Wu YJ, Liu YC, Liao CY (2021). A comparative study to evaluate CT-based semantic and radiomic features in preoperative diagnosis of invasive pulmonary adenocarcinomas manifesting as subsolid nodules. Sci. Rep..

[27] Yang Y, Feng X, Chi W (2012). Deep learning aided decision support for pulmonary nodules diagnosing: A review. J. Thorac. Dis..

[28] Lee HJ, Lee CH, Jeong YJ (2012). IASLC/ATS/ERS international multidisciplinary classification of lung adenocarcinoma: Novel concepts and radiologic implications. J. Thorac. Imaging.

[29] Gong J, Liu JY, Jiang YJ (2018). Fusion of quantitative imaging features and serum biomarkers to improve performance of computer-aided diagnosis scheme for lung cancer: A preliminary study. Med. Phys..

[30] Wang T, She Y, Yang Y (2022). Radiomics for survival risk stratification of clinical and pathologic stage IA pure-solid non-small cell lung cancer. Radiology.

[31] Parmar C, Grossmann P, Bussink J (2015). Machine learning methods for quantitative radiomic biomarkers. Sci. Rep..

[32] Zhang T, Wang Y, Sun Y (2021). High-resolution CT image analysis based on 3D convolutional neural network can enhance the classification performance of radiologists in classifying pulmonary non-solid nodules. Eur. J. Radiol..

[33] Pasini G, Bini F, Russo G (2022). matRadiomics: A novel and complete radiomics framework, from image visualization to predictive model. J Imaging.

[34] Qu H, Zhai H, Zhang S (2023). Dynamic radiomics for predicting the efficacy of antiangiogenic therapy in colorectal liver metastases. Front. Oncol..

[35] van Timmeren JE, Cester D, Tanadini-Lang S (2020). Radiomics in medical imaging-"how-to" guide and critical reflection. Insights Imaging.

