# Supplementary material for: Comparison and fusion prediction model for lung adenocarcinoma with micropapillary and solid pattern using clinicoradiographic, radiomics and deep learning features
Source: Sci Rep. 2023 Jun 8;13:9302. doi: 10.1038/s41598-023-36409-5 (PMC10250309; doi:10.1038/s41598-023-36409-5)
Supplement: Supplementary file 1 — Supplementary Information. [file 41598_2023_36409_MOESM1_ESM.docx]

**Supplementary Material**

**Supplementary Appendix 1.** The initial settings used in FeAture Explorer Pro for the feature extraction process

The following parts were involved in initial settings used in FeAture Explorer Pro:

1. Bincount: the number of bin is 20 when making histogram and discrete image grayscale.

2. Interpolator: 'sitkBSpline' is a difference method of resampling.

3. Resampled Pixel Spacing: the default parameter was used to resample the parameter, resample the pixel spacing. List of 3 floats (>= 0), sets the size of the voxel in (x, y, z) plane when resampling. A value of 0 is replaced with the spacing for that dimension as it is in the original (non-resampled) image or mask. For example, to perform only in-plane resampling, the x and y values alone should be edited (e.g.: [2,2,0]). In-plane resolution is always relative to image acquisition plane (i.e. axial, coronal or sagittal).

4. weighting Norm: a default parameter indicates which normal should be used when applying distance weighting. Applies no weighting, mean of values calculated on separate matrices is returned.

5. GeometryTolerance: 0.03 is a value of roi and focus fit determining the tolerance used by SimpleITK to compare origin, direction and spacing between image and mask.

6. Normalize: Z-Score image normalization was adopted before resampling. We normalized all exported images to a standard normal distribution before radiomics feature extraction using the following equation: f(χ)= 1000∗ (χ −µ_χ_)/σ_χ_, where µ_χ_ and σ_χ_ denote the mean and standard deviation of the image intensity, respectively.

7. Force2D is set to true to force a by slice texture calculation Dimension that identifies the ‘slice’ can be defined in.

**Supplementary Tables**

**Table S1.** Demographics and the clinical information of lung ADC with MPP/SOL pattern of training and validation sets

|  | Training set  (n=360) | Testing set  (n=154) | Mann-Whitney U value/*t* value/χ^2^ | *p* value |
| --- | --- | --- | --- | --- |
| Age (Mean SD, year) | 59.5±9.9 | 59.3±10.3 | -0.214^t^ | 0.831 |
| Gender (M/F) | 162/198 | 66/88 | 0.201^a^ | 0.654 |
| Smoking History(no/yes) | 298/62 | 124/30 | 0.374^a^ | 0.541 |
| Family History (no/yes) | 349/11 | 151/3 | 0.499^a^ | 0.480 |
| Serum CEA value(ng/ml) | 2.1 (1.4,3.5) | 2.2(1.3,3.2) | 26684.500^c^ | 0.502 |
| Serum CYFRA21-1 value  (ng/ml) | 2.2(1.7,2.7) | 2.1(1.6,2.8) | 27481.000 ^c^ | 0.877 |
| Serum NSE value (ng/ml) | 16.1(13.5,20.6) | 16.6(14.6,19.0) | 26580.000 ^c^ | 0.460 |
| CEA (negative/positive) | 59/301 | 22/132 | 0.359^a^ | 0.549 |
| CYFRA211(negative/positive) | 49/311 | 22/132 | 1.082^a^ | 0.299 |
| NSE (negative/positive) | 178/182 | 85/69 | 1.427^a^ | 0.233 |
| Tumor Location |  |  | 2697.92^c^ | 0.616 |
| RUL | 103 | 43 |  |  |
| RML | 55 | 21 |  |  |
| RLL | 109 | 60 |  |  |
| LUL | 28 | 12 |  |  |
| LLL | 65 | 18 |  |  |

**Note:** Data are mean ± standard deviation or median with interquartile range in parentheses and numbers of patients; RUL: right upper lobe; RML: right middle lobe; RLL: right lower lobe; LUL: left upper lobe; LLL: left lower lobe; " a " represents Chi-square test, "-" represents Fisher's exact tests, "c" represents Mann-Whitney U test, "t" represents Student t test.

**Supplementary Figures**

**Figure S1
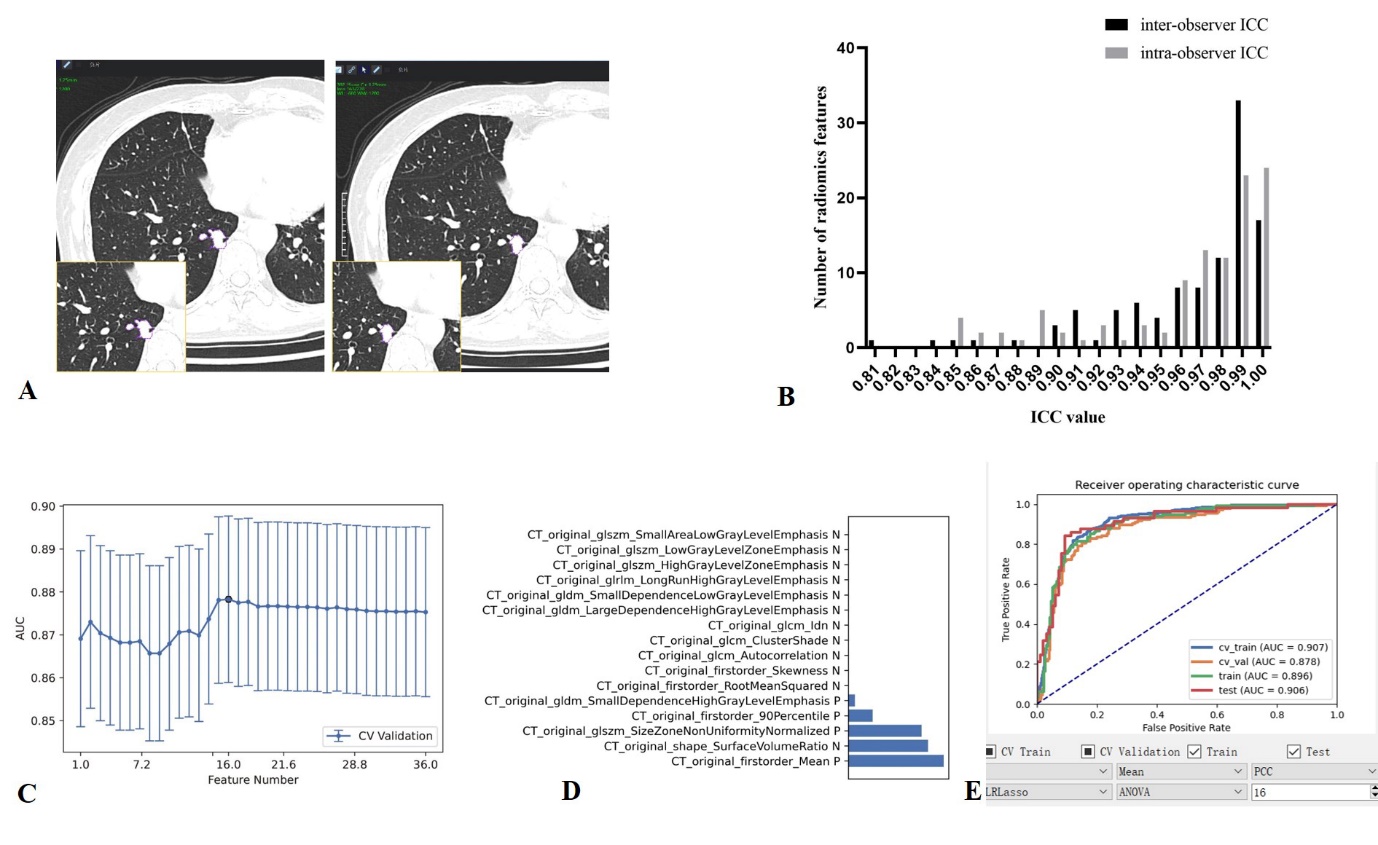
**

**Figure. S1. a-e VOI segmentation, radiomics features extraction and selection and model construction.**

Radiomics features were extracted from the manually annotated VOI implemented with in-house software(**a**). The stable features with ICC >0.80 were remained for subsequent analysis(**b**) and the finally 16 features were selected with the aid of FeAture Explorer Pro (FAE) software(**c**) in the process of ANOVA and with LASSO according to " one-standard error " rule. The weight of feature coefficients of the candidate 5 features is exhibited with the blue barchart, and " P" and " N" after the features′ name indicates the positive and negative correlation, respectively(**d**). The model′s ROC curve in different dataset was plotted ("test" and "train" represent testing and training set, respectively) (**e**).

**Figure S2**


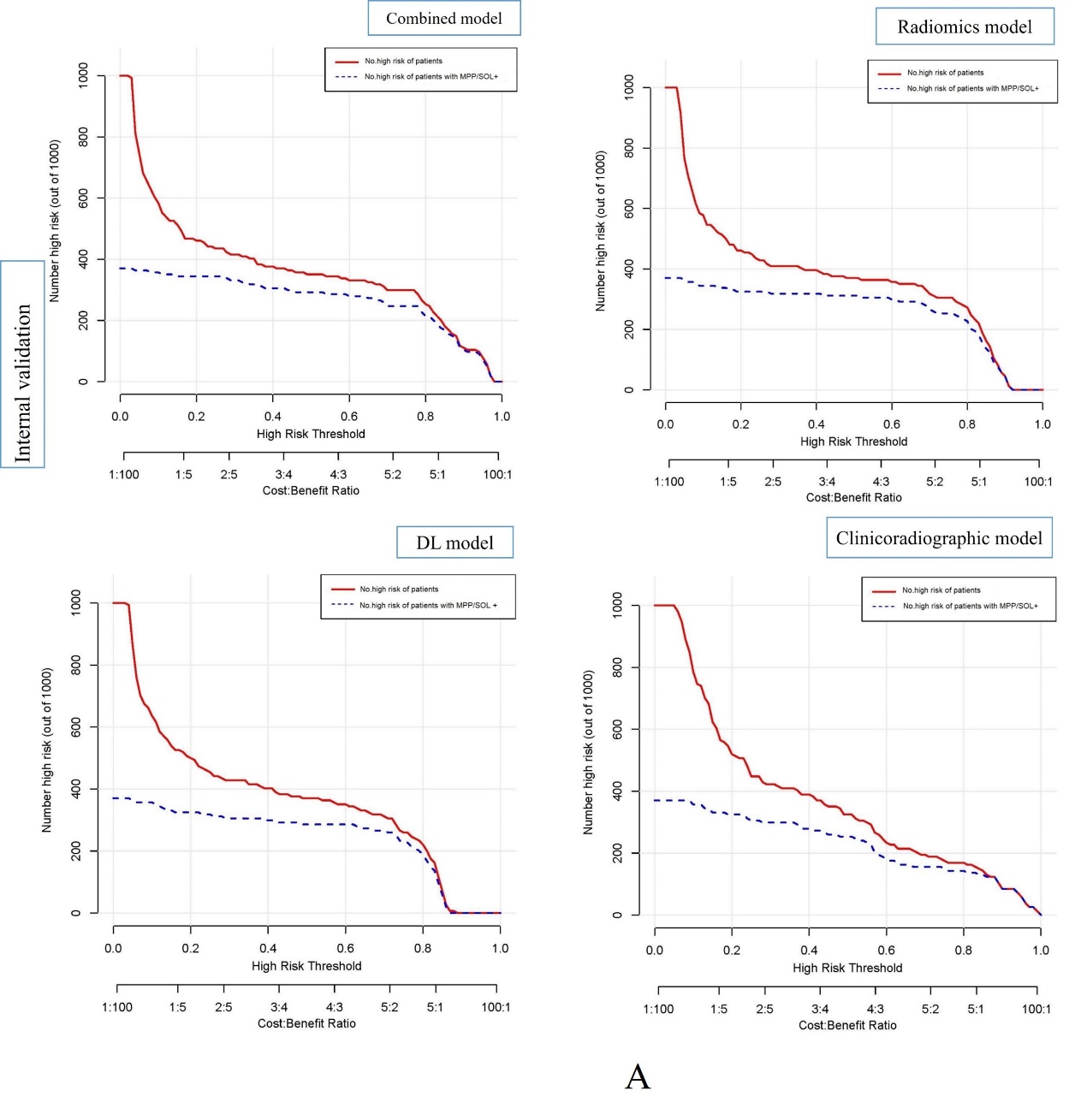


**
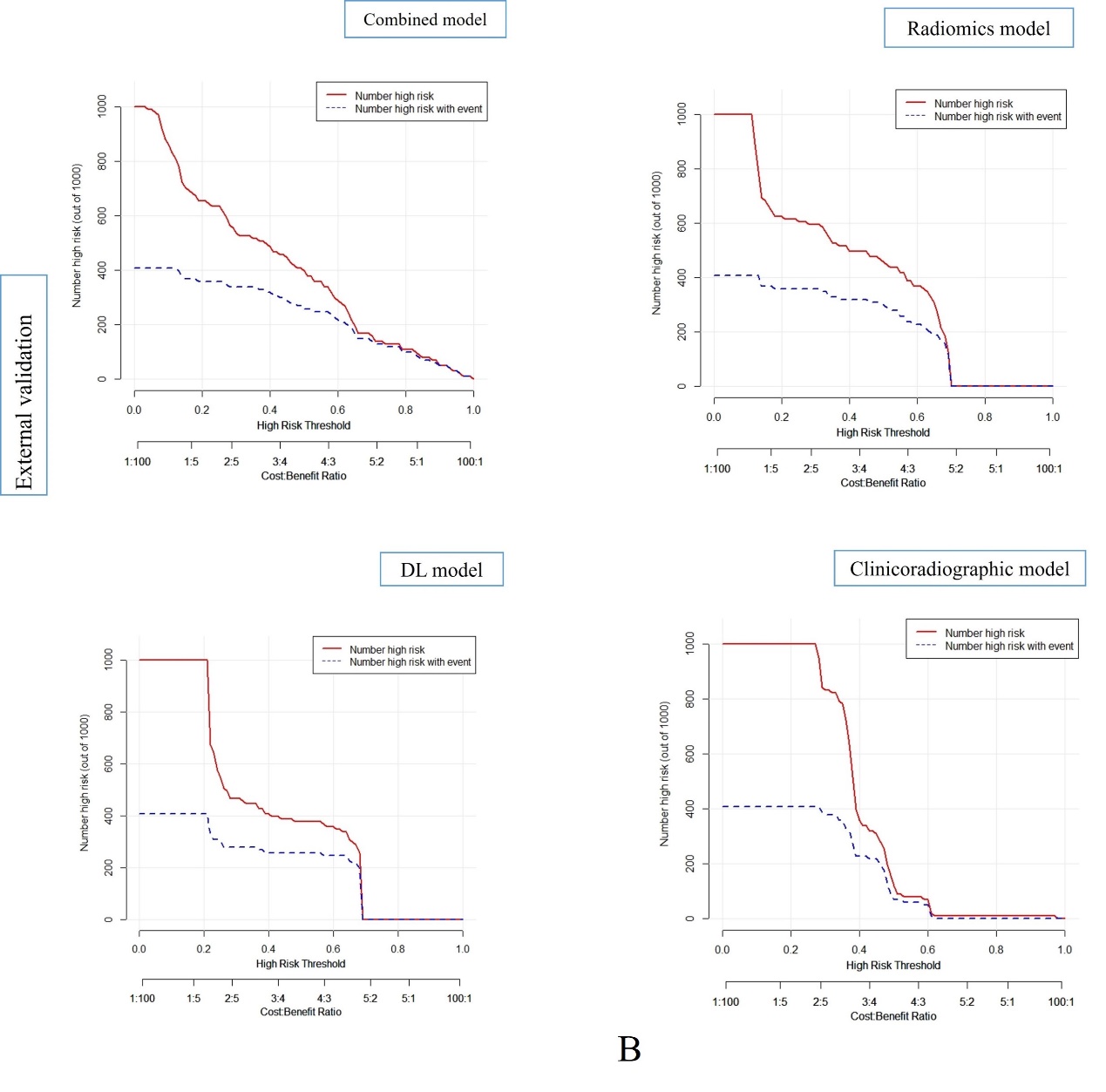
**

**Figure. S2.** **Clinical impact curve for the four models in internal validation and external validation set.**

No. means the number of. Of 1000 patients, the heavy red line represents the total number who would be deemed high risk for each risk threshold. The blue dashed line shows how many cases of those true positive cases of patients at the same risk threshold. When the threshold probability is greater than 80% of the prediction score probability value(A) in internal validation set and greater than 60% of the prediction score probability value(B) in external validation set, the four models determines that the high-risk population of MPP/SOL+ is highly matched with the actual population of MPP/SOL+(A), which indicates the high clinical efficiency of the four models.


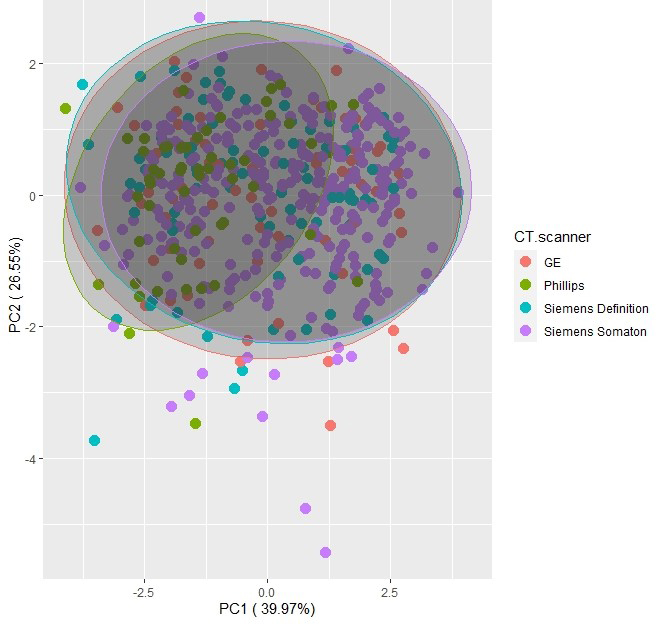


**Figure S3. PCA for radiomics features collected from different CT scanners.**
